# microRNA-29a functions as a tumor suppressor in nasopharyngeal carcinoma 5-8F cells through targeting VEGF

**DOI:** 10.22038/ijbms.2019.33818.8049

**Published:** 2019-05

**Authors:** Qingyuan Shi, Jinhua Dai, Lizhen Huang

**Affiliations:** 1 Department of Otorhinolaryngology, HwaMei Hospital, University of Chinese Academy of Sciences (Ningbo No.2 Hospital), Ningbo, Zhejiang 315010, P.R.China; 2 Department of Clinical Laboratory, HwaMei Hospital, University of Chinese Academy of Sciences (Ningbo No.2 Hospital), Ningbo, Zhejiang 315010, P.R.China

**Keywords:** 5-8F cell, miR-29a, Nasopharyngeal carcinoma- (NPC), PI3K/AKT and JAK/STAT- pathways, VEGF

## Abstract

**Objective(s):**

microRNA-29 (miR-29) family miRNAs have been mentioned as tumor suppressive genes in several human cancers. The purpose of this study was to investigate the function of miR-29a in nasopharyngeal carcinoma (NPC) cells.

**Materials and Methods:**

Human NPC cell line 5-8F was transfected with mimic, inhibitor or scrambled controls specific for miR-29a. Subsequently, cell viability, migration, apoptosis and expression changes of VEGF were assessed by trypan blue staining, MTT assay, transwell assay, flow cytometry, Western blot and RT-qPCR. TargetScan online database was used to predict the targets of miR-29a, and luciferase reporter assay was carried out for testing the targeting relationship between VEGF and miR-29a. Western blot analysis was performed to determine the expression changes of core proteins in PI3K/AKT and JAK/STAT pathways.

**Results:**

Overexpression of miR-29a suppressed 5-8F cells viability and relative migration, but increased apoptotic cell rate. Consistently, Bcl-2 was downregulated, Bax was upregulated, and caspase-3 and -9 were cleaved by miR-29a overexpression. VEGF was a target gene of miR-29a. Besides, VEGF silence exerted similar effects like miR-29a, as the viability and migration were repressed and apoptosis was induced. Finally, we found that PI3K/AKT and JAK/STAT pathways were deactivated by miR-29a or VEGF silence.

**Conclusion:**

These findings highlighted the tumor suppressive effects of miR-29a on NPC cells, as its overexpression inhibited 5-8F cells viability, migration, and induced apoptosis. miR-29a exerted tumor suppressive functions might be via targeting VEGF and deactivating PI3K/AKT and JAK/STAT pathways.

## Introduction

Nasopharyngeal carcinoma (NPC) is a distinctive type of head and neck cancer with low-frequency incidence worldwide, but it is a high risk cancer in Southeast Asia and Southern China, especially in Guangdong province (1, 2). The extract etiology of NPC remains unclear. EB-virus infection, environmental influences and heredity have been recognized as main risk factors of NPC (3). The therapeutic treatment of NPC in clinic includes surgery, chemotherapy and radiotherapy, which will achieve satisfactory outcomes (4). However, survival rates are declined with increasing tumor growth and distant metastases of NPC patients with advanced disease stage (5). Development of novel treating strategies focused on repressing NPC cells growth and metastasis is still required for improving the survival of NPC. 

microRNAs (miRNAs) are small non-coding RNAs with length approximately 22 nt. miRNAs are able to participate in multiple physiological and pathological processes through regulating target genes post-transcriptionally (6). Nowadays, miRNAs’ function in tumor cells has been investigated extensively**. **According to their function, some of them have been identified as oncogenic miRNAs or tumor-suppressive miRNAs (7). Additionally, detection of miRNA expression profiling is showing promise in clinical diagnose and prognosis prediction of human cancers (8). 

miR-29 family consists of miR-29a, miR-29b and miR-29c**, **which have been reported as key regulators in tumor cells proliferation, differentiation, apoptosis, migration and invasion (9). Aberrant expression of miR-29 has been found in many human cancers, including acute myeloid leukemia (10), gastric cancer (11), colorectal cancer (12) and osteosarcomas (13). Besides, it has been mentioned as a tumor suppressive gene in NPC, as its expression is gradually increased during the development of NPC (14, 15)**. **Meanwhile, p53 signaling and expression of immunoinhibitory molecule**s** can be significantly regulated by miR-29 (14, 15). Low plasma levels of miR-29a predicted a poor prognosis of NPC (16). A later functional experiment reported that miR-29a inhibited the proliferation and promoted apoptosis of NPC CNE-1 cells (17), showing tumor suppressive function**s.** Of contrast, another *in vitro* study proposed miR-29a as a NPC promoting gene by enhancing tumor cells migration and invasion (18). 

This study performed in human NPC 5-8F cells aimed to study the exact role of miR-29a in NPC cells growth and migration. A previous study has demonstrated that, miR-29a suppressed gastric cancer cells growth and invasion via targeting VEGF (19). Herein, we attempted to reveal whether miR-29a functioned to 5-8F cells also via regulating VEGF. Besides, PI3K/AKT and JAK/STAT pathways have now been extensively studied with investigations determining their role in carcinogenesis and the potential use of blocking these two signaling in cancer treatment (20, 21). Thus, in this study the effects of miR-29a and VEGF on the activation of PI3K/AKT and JAK/STAT pathways were studied for further explanation of the anti-tumor role of miR-29a. This study will be helpful for understanding miR-29a’s function. 

## Materials and Methods

***Cell culture***

Human NPC cell line (5-8F), a kind gift from Yixin Zeng (Cancer Center, Sun Yat-sen University, Guangzhou, China), was cultured in RPMI-1640 medium (HyClone, Logan City, Utah, USA) supplementary with 10% fetal bovine serum (FBS, HyClone). The cells were cultured at 37 ^°^C in a humidified atmosphere of 5% CO_2_.

***Cell transfection***

miR-29a mimic, miR-29a inhibitor and the corresponding scrambled controls, namely Scramble and NC, were synthesized by GenePharma (Shanghai, China). The full-length of VEGF was inserted into pcDNA3.1 plasmid (Invitrogen, Carlsbad, CA, USA) for construction of VEGF expression vector. VEGF shRNA plasmid was purchased from Santa Cruz Biotechnology (Santa Cruz, CA, USA). Empty pcDNA3.1 plasmid and the non-targeting shRNA were used as negative controls respectively. Transfection was performed when the cells in 6-well plates were reached 50% confluence by using Lipofectamine 3000 reagent (Invitrogen). After 48 hr of transfection, cells were collected and the transfection efficiency was tested by RT-qPCR and/or Western blot. 

**Figure 1:**
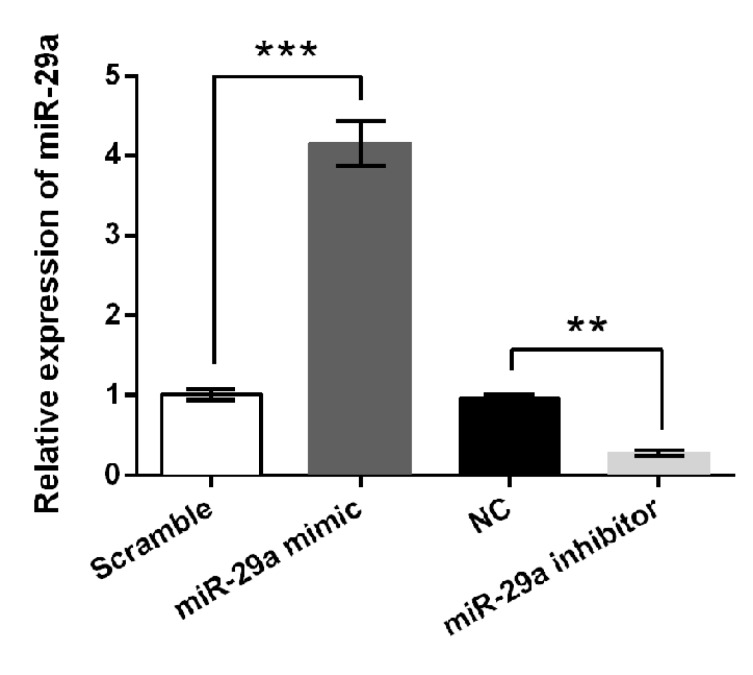
The expression changes of miR-29a after transfection. RT-qPCR analysis for detection of miR-29a after 5-8F cells were transfected with miR-29a mimic, miR-29a inhibitor or corresponding controls. ** *P<*0.01; *** *P<*0.001

**Figure 2:**
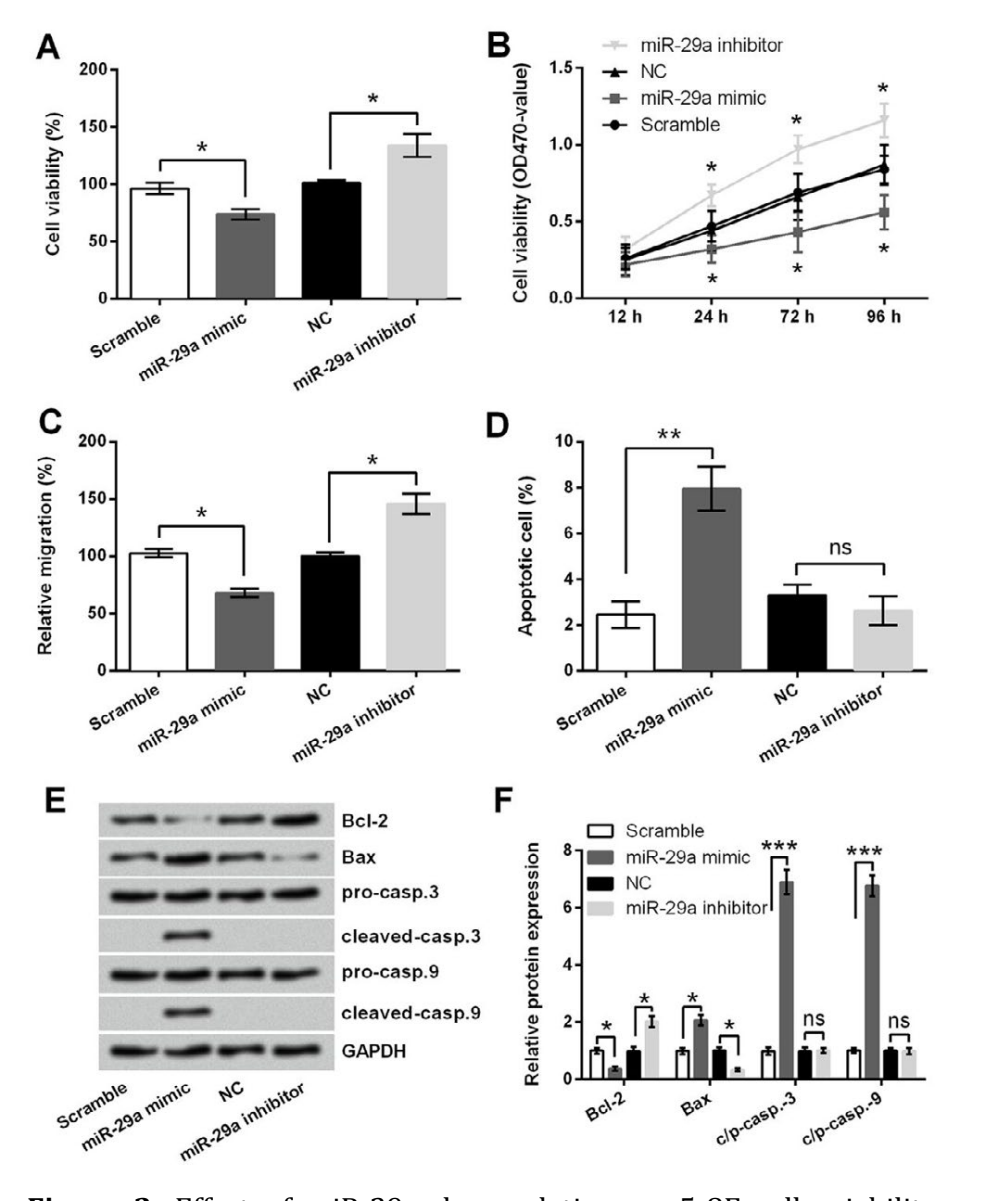
Effect of miR-29a dysregulation on 5-8F cells viability, migration and apoptosis. 5-8F cells were transfected with miR-29a mimic, miR-29a inhibitor or corresponding controls. Cell viability was respectively assessed by (A) trypan blue staining and (B) MTT assay. (C) Migration, (D) apoptotic cell rate, and (E-F) expression changes of apoptosis-related proteins were respectively assessed by transwell assay, flow cytometry, and Western blot. ns, no significant; * *P<*0.05; ** *P<*0.01; *** *P<*0.001

**Figure 3:**
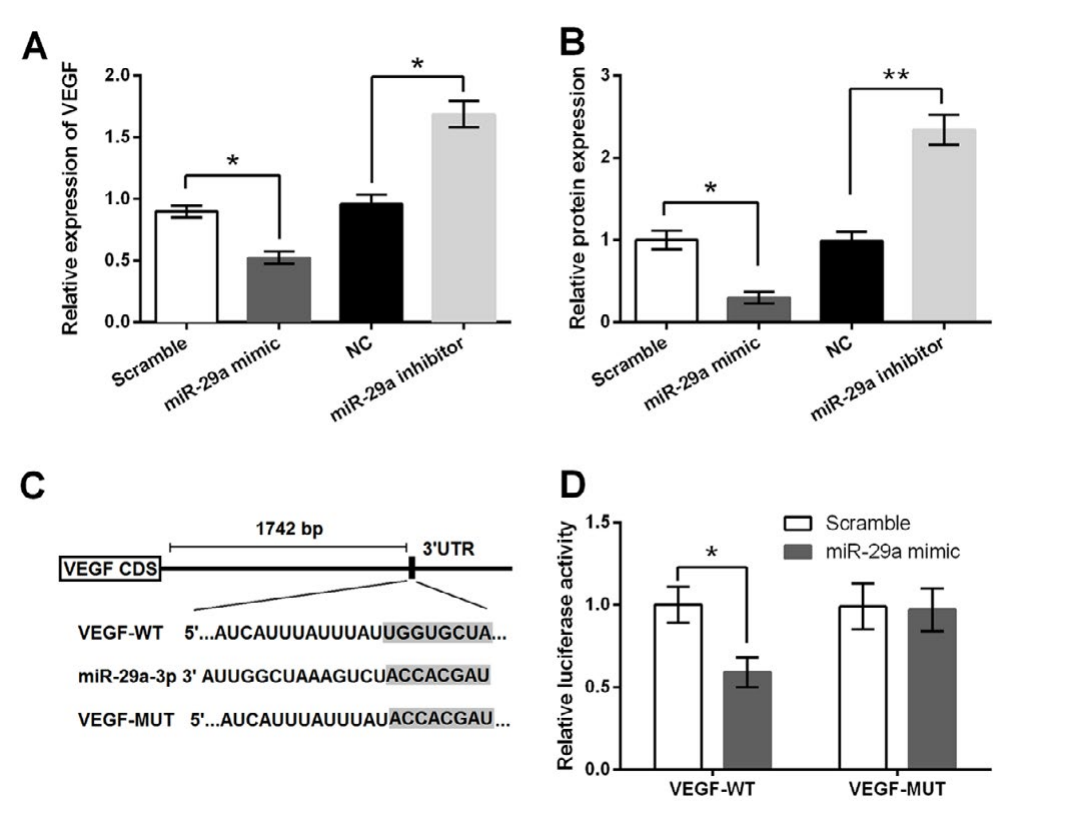
Effect of miR-29a on vascular endothelial growth factor (VEGF) expression. 5-8F cells were transfected with miR-29a mimic, miR-29a inhibitor or corresponding controls. (A) mRNA and (B) protein expression levels of VEGF were measured by RT-qPCR and Western blot respectively. (C) The predicted consequential pairing of VEGF 3’UTR and miR-29a. (D) Luciferase reporter assay was carried out for testing the targeting relationship between VEGF and miR-29a. * *P<*0.05; ** *P<*0.01

**Figure 4:**
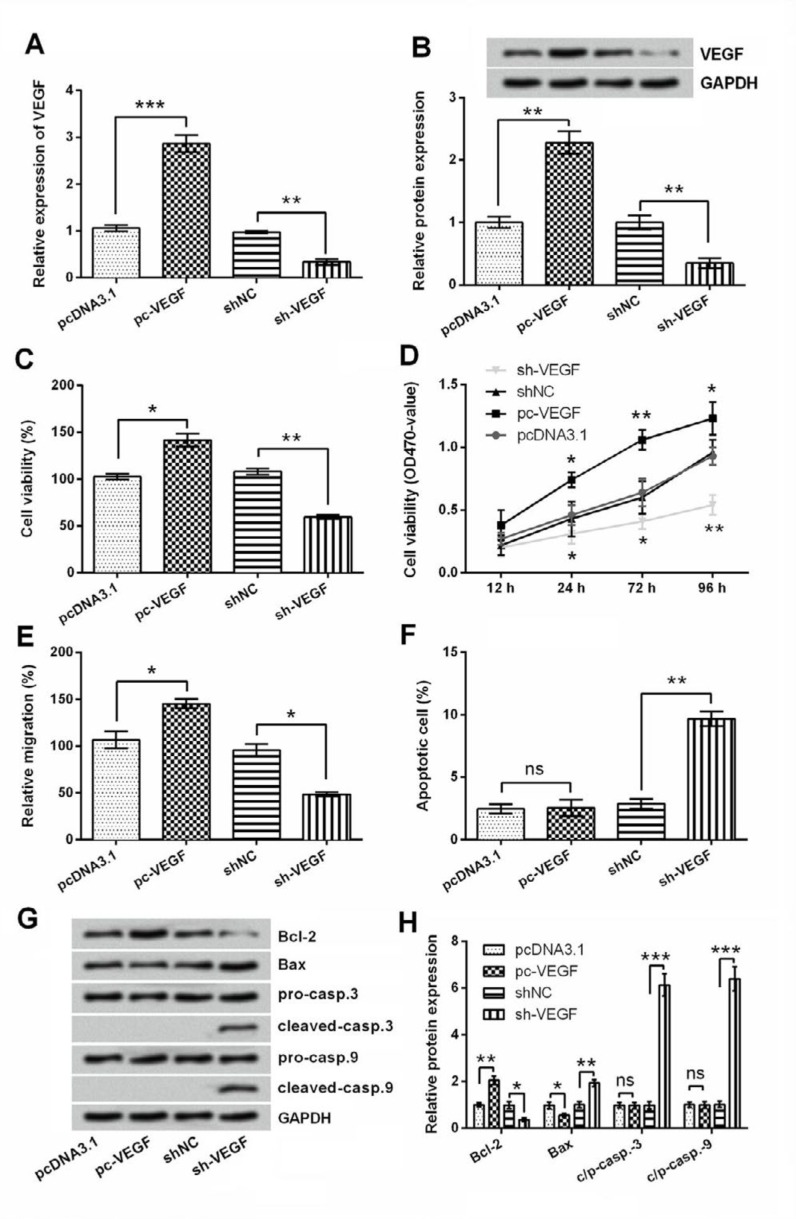
Effect of vascular endothelial growth factor (VEGF) dysregulation on 5-8F cells viability, migration and apoptosis. 5-8F cells were transfected with pc-VEGF, sh-VEGF or corresponding controls. (A) mRNA and (B) protein levels of VEGF were measured by RT-qPCR and Western blot respectively. Cell viability was measured by (C) trypan blue staining and (D) MTT assay. (E) Migration, (F) apoptotic cell rate, and (G-H) expression changes of apoptosis-related proteins were respectively assessed by transwell assay, flow cytometry, and Western blot. ns, no significant; * *P<*0.05; ** *P<*0.01; *** *P<*0.001

**Figure 5:**
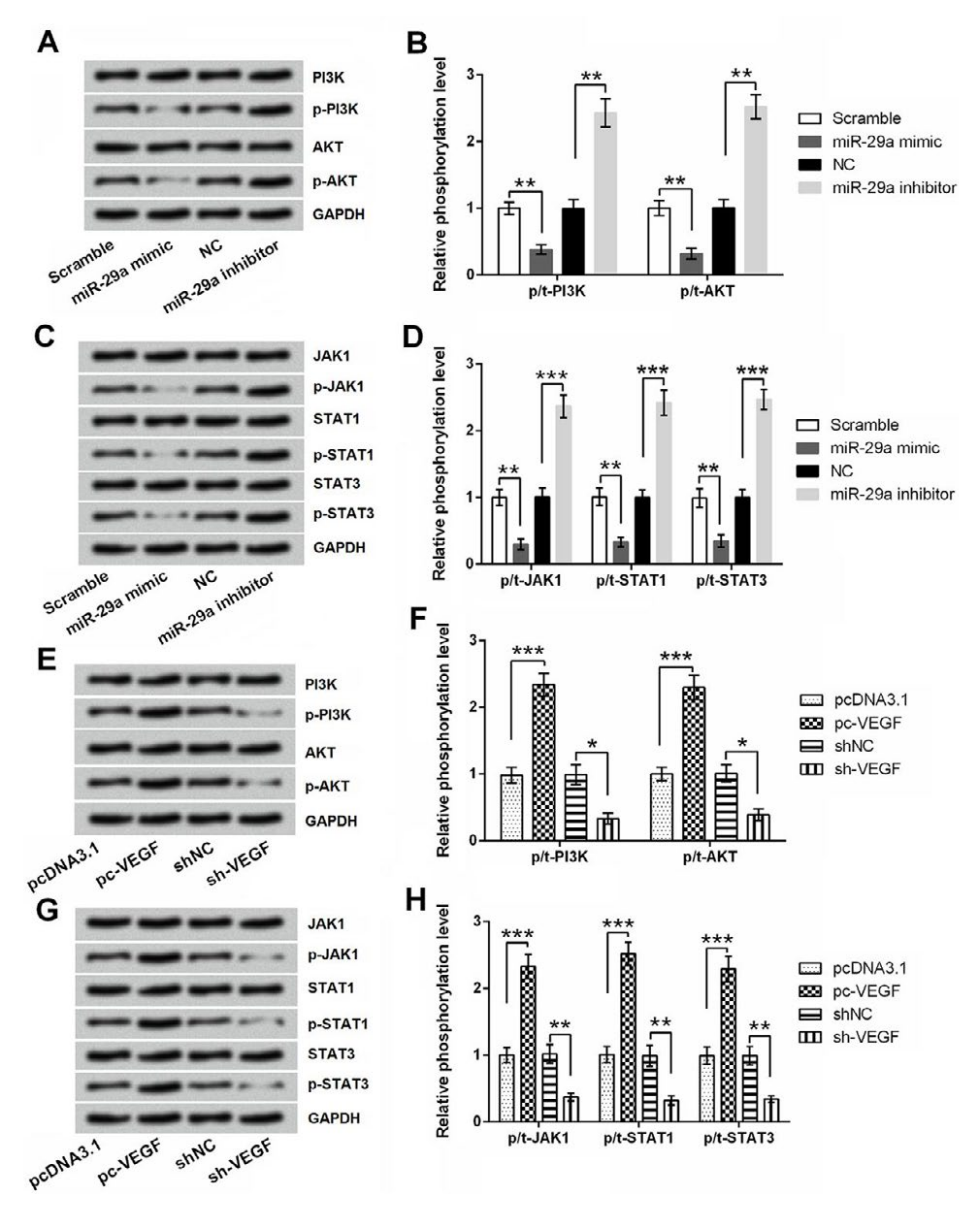
Effect of miR-29a and vascular endothelial growth factor (VEGF) dysregulation on the activation of phosphatidylinositol 3-kinase/otein-serine-thre-onine kinase (PI3K/AKT) and Janus kinase/signal transducer and activator of transcripti (JAK/STAT) pathways. 5-8F cells were transfected with miR-29a mimic, miR-29a inhibitor or corresponding controls. Expression changes of core proteins in (A-B) PI3K/AKT and (C-D) JAK/STAT pathways were tested by Western blot. 5-8F cells were transfected with pc-VEGF, sh-VEGF or corresponding controls. Expression changes of core proteins in (E-F) PI3K/AKT and (G-H) JAK/STAT pathways were tested by Western blot. * *P<*0.05; ** *P<*0.01; *** *P<*0.001

***Trypan blue staining***

The transfected cells were seeded in 24-well plates with a density of 1 × 10^5^ cells/well for 48 hr of incubation. The cells were trypsinized and stained by 0.4% trypan blue (Solarbio, Beijing, China) for 5 min, after which the viable cells were counted under a microscope (CX23, Olympus, Tokyo, Japan). 

***CCK-8 assay***

Cell viability was also detected by 3-(4,5-dimethyl-2-thiazolyl)-2,5-diphenyl-2-H-tetrazolium bromide (MTT) assay. The transfected cells were seeded in 96-well plates with a density of 5×10^5^ cells/well. The cells were then incubated at 37 ^°^C for 12-96 hr. MTT solution (Sigma-Aldrich, St Louis, MO, USA) with a final concentration of 5 mg/ml was added into each well, and the plates were incubated at 37 ^°^C for another 4 hr. The culture medium was removed, and 150 μl dimethyl sulfoxide (DMSO, Sigma-Aldrich) was added into each well. The plates were shaken in an ELISA reader (Bio-Rad, Hercules, CA, USA) for 10 min, after which the absorbance (OD-value) at a wavelength of 470 nm was measured. 

***Migration assay***

The transfected cells were resuspended in 200 μl serum-free medium and placed into the upper chamber of a 24-well transwell cell culture chamber (Coster, Cambridge, MA) equipped with a filter membrane with 8-µm pores. The lower chamber was filled with 600 μl complete medium. After 48 hr of incubation at 37°C, the cells were fixed with methanol. Cells in the upper chamber were removed by cotton swab, and cells in the lower chamber were stained by crystal violet (Beyotime, Shanghai, China). The stained cells were counted under the microscope (CX23, Olympus, Tokyo, Japan). 

***Apoptosis assay***

Cell apoptosis was detected after transfection by using Annexin V-PE Apoptosis Detection Kit (Beyotime). In brief, 1×10^5^ transfected cells were resuspended in 195 µl Annexin V-PE binding buffer. Then, 5 µl Annexin V-PE was mixed in the cell suspension, and followed by a 15 min of incubation at room temperature in the dark. The samples were then determined by a flow cytometer (Beckman Coulter, USA). 

***Prediction of miR-29a-targeting genes***

TargetScan online database (http://www.targetscan.org/vert_71/) was used to predict target genes for miR-29a. 

***Luciferase reporter assay***

The predicted binding site in 3’UTR of VEGF was amplified by PCR and was inserting into pmiR-Report vector (Ambion, Austin, TX, USA) for construction of a reporter vector VEGF-WT. The predicted binding sites were replaced by the unpaired bases and were referred as VEGF mutated type (VEGF-MUT). These vectors were co-transfected with miR-29 mimic or Scramble into 293T cells. After 48 hr of transfection with the help of Lipofectamine 3000 reagent, luciferase assay was carried out for testing luciferase activity by using Dual-Luciferase reporter assay system (Promega, Madison, WI, USA) as previously described (22). 

***RT-qPCR***

Total RNAs in the transfected cells were extracted by the TRIzol reagent (Invitrogen). The expression of miR-29a was tested by using the Mir-X™ miRNA First Strand Synthesis Kit and Mir-X™ miRNA qRT-PCR SYBR^®^ Kit (Takara Biotechnology, Dalian, China). The expression of VEGF was tested by using the Transcriptor First Strand cDNA Synthesis Kit and FastStart Universal SYBR Green Master (Roche, Basel, Switzerland). The expression of miR-29a was normalized to U6, and VEGF was normalized to GAPDH. Data were analyzed according to the classic 2^−ΔΔCt^ method. The primer sequences were listed as follows. miR-29a: forward 5’-GGGTAGCACCATCTGAAAT-3’, reverse 5’-CAGTGCGTGTCGTGGAGT-3’; U6: forward 5’-CTCGCTTCGGCAGCACA-3’, reverse 5’-AACGCTTCACGAATTTGCGT-3’; VEGF: forward 5’-CAACATCACCATGCAGATTATGC-3’, reverse 5’-CCCACAGGGATTTTCTTGTCTT-3’; GAPDH: forward 5’-GAAGGTGAAGGTCGGAGTC-3’, reverse 5’- GAAGATGGTGATGGGATTTC-3’. 

***Western blot***

Total proteins in the transfected cells were isolated by 1% Triton X-100 and 1 mM PMSF (pH 7.4) over ice for 30 min. The protein concentration of the whole-cell extract was tested by BCA™ Protein Assay Kit (Pierce, Appleton, WI, USA). Proteins were separated by SDS-PAGE and transferred onto polyvinylidene fluoride (PVDF) membrane. The membranes were blocked in 5% nonfat dry milk for 1 hr at room temperature, followed by incubation with primary antibodies at 4 ^°^C overnight. Antibodies specific for Bcl-2 (ab692), Bax (ab32503), pro-caspase-3 (ab32499), cleaved-caspase-3 (ab2302), pro-caspase-9 (ab2013), cleaved-caspase-9 (ab2324), VEGF (ab52917), PI3K (ab86714), p-PI3K (ab182651), AKT (ab8805), p-AKT (ab38449), JAK1 (ab47435), p-JAK1 (ab215338), STAT1 (ab3987), p-STAT1 (ab30645), STAT3 (ab119352), p-STAT3 (ab76315), and GAPDH (ab181602) were all purchased from Abcam (Cambridge, MA, USA). The membranes were then incubated with the secondary antibodies for 1 hr at room temperature. The protein bands were developed by chemiluminescence and autoradiography, and the intensity of the bands was quantified using Image Lab™ Software (Bio-Rad Laboratories, Hercules, CA, USA).

***Statistical analysis***

Data was presented as mean±SD from three repeated experiments. Statistical difference between groups was tested by ANOVA in SPSS 19.0 software (SPSS Inc., Chicago, IL, USA). Statistical difference was identified as *P*-value of <0.05. 

## Results

***miR-29a was overexpressed or suppressed after transfection***

The expression levels of miR-29a in 5-8F cells were altered by transfection. RT-qPCR data in Figure 1 showed that the expression level of miR-29a was significantly increased in miR-29a mimic transfected cells as compared to Scramble control (*P*<0.001). Meanwhile, miR-29a expression was significantly decreased in miR-29a inhibitor transfected cells when compared to NC transfected cells (*P*<0.01). These data suggested that miR-29a was successfully overexpressed or suppressed in 5-8F cells by transfection. 

***Increased miR-29a level inhibited viability and migration, but induced apoptosis of 5-8F cells***

The changes in cell viability, migration and apoptosis were tested respectively post-transfection to evaluate the functions of miR-29a in 5-8F cells. Figure 2A-2C showed that cell viability and migration were both significantly reduced in miR-29a mimic group, and increased in miR-29a inhibitor group, as compared with their corresponding controls (all* P*<0.05). Figure 2D showed that apoptotic cell rate was increased in miR-29a mimic group when compared to Scramble group (*P*<0.01). However, no significant changes in apoptotic cell rate between miR-29a inhibitor group and NC group were observed (*P*<0.05), since the rate in NC group is already very low. Consistently, Western blot results in Figure 2E-2F displayed that Bcl-2 was downregulated (*P*<0.05)**,** Bax was upregulated (*P*<0.05) and caspase-3 and -9 were remarkably cleaved (*P*<0.001) in miR-29a mimic group. Meanwhile, Bcl-2 was upregulated (*P*<0.05), Bax was downregulated (*P*<0.05) and caspase-3 and -9 were unchanged (*P*>0.05) in miR-29a inhibitor group. 

***VEGF was a target gene of miR-29a***

Previous studies have mentioned VEGF as a downstream effector of miR-29a in inhibition of gastric cancer (19) and osteoarthritis (23). Present work studied the regulatory relationship between miR-29a and VEGF in 5-8F cells to see whether miR-29a contributed to NPC cells growth and migration also via regulating VEGF. By performing RT-qPCR and Western blotting, we found that both the mRNA and protein levels of VEGF were low expressed in miR-29a mimic group, while were highly expressed in miR-29a inhibitor group, as compared to their corresponding controls (Figure 3A-3B). These data indicated that VEGF expression was negatively regulated by miR-29a. Next, we studied whether VEGF was a downstream target for miR-29a. TargetScan online database predicted that VEGF could directly bind with miR-29a (Figure 3C). This prediction was further confirmed by luciferase reporter assay, suggesting VEGF was a target gene of miR-29a (Figure 3D). 

***Decreased VEGF level inhibited viability and migration, but induced apoptosis of 5-8F cells***

Next, the functions of VEGF in 5-8F cells were tested by detection of cell viability, migration and apoptosis after transfection of pc-VEGF, sh-VEGF or corresponding controls. Transfection efficiency was verified by RT-qPCR and Western blotting. Figure 4A and 4B showed that VEGF overexpressing- and VEGF silencing-cells were successfully obtained. More importantly, transfection of cells with pc-VEGF significantly increased cell viability and migration (both* P*<0.05, Figure. 4C-4E), while have no impacts on apoptotic cell rate (*P*>0.05, Figure. 4F). Of contrast, transfection of cells with sh-VEGF significantly decreased viability (*P*<0.01) and migration (*P*<0.05), but increased apoptotic cell rate (*P*<0.01). Western blotting results indicated that Bcl-2 was up-regulated (*P*<0.01), Bax was down-regulated (*P*<0.05) and caspase-3 and -9 were unchanged (*P*>0.05) in pc-VEGF transfected cells (Figure 4G-4H). Meanwhile, Bcl-2 was down-regulated (*P*<0.05), Bax was up-regulated (*P*<0.01) and caspase-3 and -9 were cleaved (*P*<0.001) in sh-VEGF transfected cells. Collectively, VEGF silence shows miR-29a-like effects on 5-8F cells. 

***Increased miR-29a level and decreased VEGF level deactivated PI3K/AKT and JAK/STAT pathways***

Finally, the expression changes of core kinases in PI3K/AKT and JAK/STAT pathways were measured to decode whether miR-29a and VEGF impacted 5-8F cells though these two signaling. Figure 5A-5D displayed that, the phosphorylation levels of PI3K, AKT, JAK1, STAT1, and STAT3 were all remarkably decreased by miR-29a mimic (*P*<0.01), while were increased by miR-29a inhibitor (*P*<0.01 or *P*<0.001). Opposite trend was observed in Figure. 5E-5H, that the phosphorylation levels of these proteins were increased by pc-VEGF (*P*<0.001), while were decreased by sh-VEGF (*P*<0.05 or *P*<0.01). The total levels of these proteins were unchanged either by miR-29 dysregulation or by VEGF dysregulation. 

## Discussion

Although the management of NPC has been largely improved in recent decades, the 5-years survival rate remains poor, due to the high recurrence and distant metastasis (24). Therefore, it is imperative to fully elucidate the underlying mechanisms involved in the growth and metastasis of NPC cells, which will provide fundamental information for the development of treating strategies. Recent literatures reported that**, **a growing number of miRNAs are implicated in the growth and migration of NPC cells, including miR-371-5p (25), miR-203a-3p (26), miR-130a-3p (27), and miR-29a (17, 18). In the current study, we also demonstrated the involvement of miR-29a in NPC cells growth and migration. Besides, *in vitro *experiments performed in 5-8F cells revealed that the functions’ of miR-29a in 5-8F cells might be via modulation of VEGF as well as PI3K/AKT and JAK/STAT pathways. 

Sporadic studies have focused on investigating the role of miR-29a in NPC, but its function in oncogenesis and development of NPC is still confusing. An* in silico *analysis has proposed that miR-29a might function as a tumor suppressive gene in the stepwise development of NPC (28), due to miR-29a could significantly mediate p53 signaling and immuneinhibition (14, 15). This was confirmed by a later study, in which miR-29a overexpression inhibited the proliferation, but promoted apoptosis of CNE-1 cells (17). Actually, the tumor suppressive role of miR-29a has been widely reported in other cancer types, like hepatocellular carcinoma (29), lung cancer (30), glioma (31), and papillary thyroid carcinoma (32). However, another* in vitro* study suggested miR-29a as an oncogenic miRNA in NPC, as miR-29a overexpression increased S18 cells migration and invasion (18). Present work performed in NPC 5-8F cell line demonstrated that miR-29a overexpression repressed the viability and migration of tumor cells, while induced apoptosis, suggesting the tumor suppressive role of miR-29a in NPC. This contradiction might be caused by different cell types used in study. 

An increasing number of literatures have demonstrated that miR-29a exerted its functions via regulating several relevant effectors in cancer, including CAV2 (33), STAT3 (17), SIRT1 (29), and NRAS (30). VEGF, a promoter of tumorigenesis, has also been mentioned as one of such downstream effectors of miR-29a (23). miR-29a lowered VEGF production and thus suppressed gastric cancer cells growth and invasion (19). Similar results were observed in this study, that miR-29a overexpression led to a down-regulation of VEGF. Luciferase reporter assay results revealed that VEGF was a target gene of miR-29a. Additionally, VEGF silence showed miR-29a-like effects on 5-8F cells, as the viability and migration were decreased, and apoptosis was induced by VEGF silence. Together, these findings suggested that miR-29a exerted anti-migratory and pro-apoptotic effects on 5-8F cells possibly via targeting VEGF. 

PI3K/AKT and JAK/STAT signaling pathways play significant roles in regulating tumor cells proliferation, apoptosis, and migration (34, 35). Previous studies have showed **a** close relationship between miR-29a and STAT. For example, in melanoma cells, miR-29a could be up-regulated by IFN-γ in STAT1-dependent signaling (36). Moreover, in NPC CNE-1 cells, miR-29a overexpression inhibited STAT3 expression, indicating STAT3 was negatively regulated by miR-29a (17). Also, miR-29 expression has been linked with the activation of PI3K/AKT pathways (32, 37). The present work demonstrated that miR-29a overexpression remarkably repressed PI3K/AKT and JAK/STAT signaling pathways. VEGF exhibited opposite results in there two signaling, showing promoting effects on the activation of PI3K/AKT and JAK/STAT pathways. Our findings suggested the tumor suppressive effect of miR-29a and the tumor promoting effect of VEGF on NPC cells might be via modulating these two signaling. 

## Conclusion

Taken together, the findings of this study highlighted the tumor suppressive effects of miR-29a on NPC cells, as its overexpression inhibited 5-8F cells viability, migration, and induced apoptosis. miR-29a exerted tumor suppressive functions might be via targeting VEGF and deactivating PI3K/AKT and JAK/STAT pathways. 
